# Environmental sustainability in sports events: specifications for responsible management

**DOI:** 10.3389/fspor.2026.1749862

**Published:** 2026-07-15

**Authors:** Michel Desbordes, Celeste d’Ettorre

**Affiliations:** Institute of Sport Sciences (ISSUL), University of Lausanne, Lausanne, Switzerland

**Keywords:** energy budget, environmental impact, sport ecology, sports events, sustainable solutions

## Abstract

Sports events play an important role in society by bringing people together, inspiring communities, and building resilience. However, as these events grow bigger and more complex, they face serious environmental challenges including carbon emissions, waste generation, and resource overuse. This research investigates how environmental sustainability can be integrated into sports event management, focusing on strategies to reduce ecological harm while maintaining operational excellence. The study combines a review of academic literature, interviews with industry professionals, and case studies of major events like the FIFA World Cup Qatar 2022 and the Paris 2024 Olympics. It identifies key environmental challenges, explores the effectiveness of sustainability tools and frameworks such as ISO 20121 and the circular economy model, and highlights the importance of collaboration among stakeholders. Findings show that making sports events more sustainable requires strong leadership, cultural changes, and innovative practices tailored to specific contexts. While zero environmental impact remains unattainable, significant reductions are possible through mindful resource usage, energy budgeting, and waste minimization. This research aims to demonstrate that sports events can serve not only as platforms for athletic excellence but also as catalysts for global environmental progress, proving that meaningful change is both necessary and achievable.

## 1 Introduction

Sport has long served as a driving force for cultural development, community engagement, and global unity. From the precision of elite athletes on the world stage to the passion of local competitions, sport embodies excellence and shared human values. These global sporting spectacles, while offering excitement and inspiration, also depend fundamentally on the Earth's abundant natural resources, water, snow, ice, grass, and thriving ecosystems. As environmental conditions become more precarious, the sustainability of these events and the landscapes that support them is increasingly called into question. This evolving reality demands urgent attention to the intricate relationship between sports and environmental health.

Climate change and environmental degradation already affect sporting activities worldwide. Rising temperatures, extreme weather events, and unpredictable snowfalls have disrupted both professional and amateur competitions. The 2022 Beijing Winter Olympics, which relied on over 90% artificial snow, exemplifies how fragile these conditions have become (1). Similarly, the increasing incidence of heat-related illnesses in summer events such as the FIFA World Cup highlights the vulnerability of athletes and spectators to a warming climate (2). These trends reveal that sport is both affected by and contributes to environmental change.

According to the United Nations Sustainable Development Goals (3), addressing climate change is a pressing priority that affects social well-being, health, and economic development (4). The sports industry, highly commercialized and globalized, plays a substantial role in this dynamic. Event organization, facility construction, and large-scale travel of fans and teams generate emissions, waste, and resource consumption (5). However, sport also represents a powerful tool for advancing sustainability. The United Nations 2030 Agenda recognizes its capacity to promote inclusion, health, gender equality, and environmental awareness (3). Through responsible management and strategic planning, sport can act as a catalyst for environmental consciousness and behavioral change.

Despite growing attention to sustainability, research on the environmental impact of sport events remains fragmented. Studies have highlighted the potential of frameworks such as ISO 20121, GRI standards, and circular economy principles to reduce environmental impact, yet their integration into sports management practices is still limited. There is a clear need for empirical research that bridges theory and application, providing event organizers with operational models for sustainable management.

This study addresses this gap by examining how environmental sustainability can be integrated into sport event management. It explores existing best practices, policies, and management tools to develop guidelines that enable event organizers to minimize ecological harm while maintaining operational efficiency and enhancing participant experience. The research analyzes sustainability strategies implemented in major international events to identify effective approaches and common barriers to their adoption.

To achieve this aim, the study applies a qualitative research design combining case study analysis and expert insights. Case studies provide the theoretical foundation for understanding sustainability within sport events, while interviews offer practical perspectives from professionals and stakeholders. The findings contribute to both academic knowledge and managerial practice by defining actionable strategies for implementing sustainability across key operational areas such as venue design, waste management, and transportation.

## 2 Materials and methods

### 2.1 Literature review

#### 2.1.1 Concept and framework of sustainability

##### 2.1.1.1 Defining sustainability

Sustainability is a multidimensional concept that balances environmental integrity, social equity, and economic viability. The Brundtland Report (6) defines sustainable development as “development that meets the needs of the present without compromising the ability of future generations to meet their own needs.” These three interdependent pillars: environmental, economic, and social sustainability, form the foundation for long-term global stability (7).

Environmental sustainability focuses on conserving biodiversity and managing natural resources responsibly (8); economic sustainability promotes inclusive prosperity within ecological limits (7); and social sustainability ensures equity, participation, and quality of life (9). Achieving sustainability therefore requires a continuous balance between these dimensions, as progress in one inevitably affects the others.

##### 2.1.1.2. Evolution of sustainability

Historically, the concept of sustainability has developed through centuries of reflection on the relationship between human activity and the natural environment. Hans Carl von Carlowitz first introduced the notion of Nachhaltigkeit (“sustainability”) in 1713, emphasizing the need to balance forest use with regeneration (10). During the 19th and 20th centuries, industrial expansion and population growth accelerated environmental degradation, revealing the finite nature of natural resources (11). A major turning point came with The Limits to Growth (43), which warned against unchecked economic growth and inspired the first United Nations Conference on the Human Environment held in Stockholm (12).

##### 2.1.1.3 Context of sustainability implementation in sport

The modern definition of sustainable development was established in *Our Common Future* (6), setting the stage for the United Nations 2030 Agenda and its 17 Sustainable Development Goals (SDGs) (3). These goals integrate environmental, economic, and social dimensions of development and recognize sport as a powerful driver for sustainability, promoting health, equality, inclusion, and climate action (3, 5).

This conceptual framework provides the foundation for examining how sustainability principles can be applied to sport management and event organization.

#### 2.1.2 Sport ecology and environmental dependency—environmental sustainability in sports event

Sport and the natural environment share a bidirectional relationship: while sport depends on natural resources such as snow, grass, and water, it also contributes to environmental degradation through energy consumption, waste generation, and carbon emissions (13). This mutual dependence defines the emerging field of *sport ecology*, which examines both how environmental conditions influence sports and how sports, in turn, affect the environment.

##### 2.1.2.1 The bidirectional relationship between sport and the environment

The concept of sport ecology is defined as “*the study of sport, the natural environment, and the bidirectional relationship between the two*” (13). It has become a recognized subdiscipline within sport management (14, 40), emphasizing the need for interdisciplinary approaches to understand and mitigate sport's ecological footprint. Sport ecology provides a scientific framework that connects environmental science with sport management, promoting data-driven strategies for sustainability. Through this lens, environmental sustainability in sport is not only about protecting the environment but also about preserving the long-term viability of sport itself (15).

Climate change has already demonstrated measurable impacts on sport. For instance, the 2018 Winter Olympics in PyeongChang relied almost entirely on artificial snow (1, 16) and extreme heat has increasingly disrupted summer competitions, posing health risks to athletes and spectators (17). These examples highlight how global warming is reshaping event calendars, training conditions, and even the geographical viability of sports.

At the same time, the organization of sport events contributes significantly to environmental pressures. Venue construction, international travel, and mass tourism generate high carbon emissions and resource depletion (18). Even small-scale sporting activities, when multiplied across million of participants, create cumulative environmental effects that cannot be ignored (14).

This interdependence positions sport as both a victim and a contributor to climate change (19). To ensure its long-term viability, sustainability should be integrated into sport management as a central strategic objective rather than treated as an optional initiative.

#### 2.1.3 Sustainability management in sport events

##### 2.1.3.1 Sports events management: an interactive system

**Figure 1:**
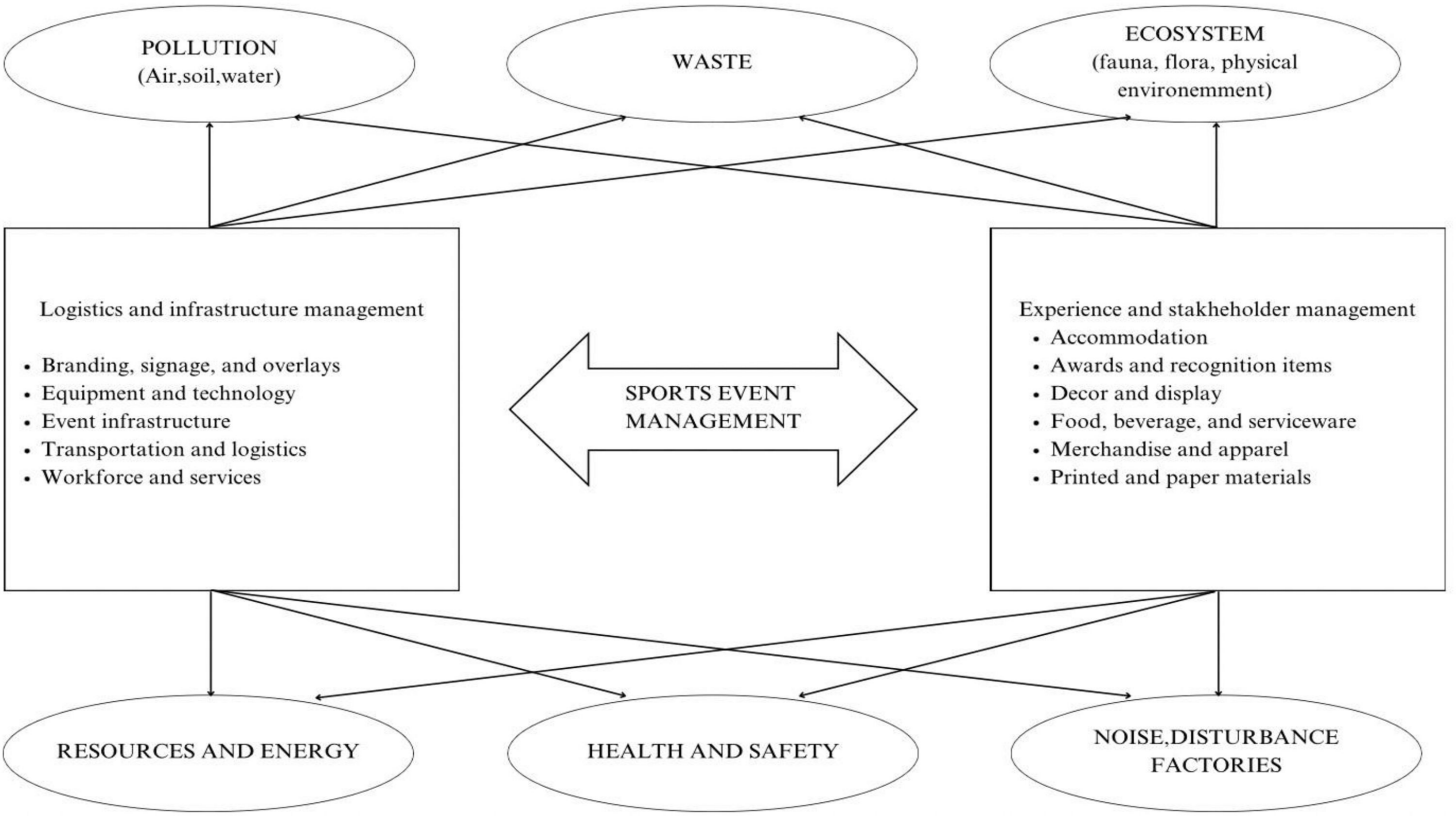
Interconnections between sports event management and environmental impacts. Source: adapted from Sorrentini (21).

Sport events are complex systems that require the coordinated interaction of multiple stakeholders, each contributing to operational, logistical, and strategic processes (20). These systems require significant material and energy resources, along with efficient organization and planning. As a result, they inevitably produce environmental impacts that need to be assessed and mitigated through sustainable management practices (21). Within this context, sustainability management has emerged as a key framework to balance environmental responsibility with event performance and stakeholder satisfaction (Figure 1).

The organizational structure of sport events reveals two main areas of intervention: logistics and infrastructure management, and stakeholder experience management. The first involves operations such as venue preparation, transportation, and technological support, activities that often result in high resource consumption, waste generation, and pollution. The second relates to the experience of participants and spectators, including accommodation, food services, and communication activities, which also contribute to environmental pressure and waste production.

##### 2.1.3.2 The strategic role of sports managers

**Figure 2:**
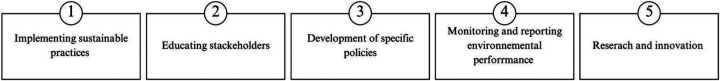
Sports management strategies and practices for supporting sustainability. Source: design based on Safarpour et al. (14).

Reducing these impacts requires a balanced approach that integrates environmental, social, and economic objectives. However, financial pressures frequently dominate decision-making, compromising sustainability principles (22). Sport managers therefore play a pivotal role. Effective sport management relies on integrating sustainability into organizational culture and daily operations. It involves actions such as using renewable energy, reducing waste, and promoting recycling, while raising awareness among athletes, staff, and spectators. The adoption of clear environmental policies, transparent evaluation systems, and collaboration with universities and local stakeholders enhances accountability and fosters innovation (14). Altogether, these measures form a comprehensive model that balances environmental, social, and economic dimensions, emphasizing sport's role in advancing ecological and social well-being (Figure 2).

#### 2.1.4 Sustainability tools and frameworks

To put sustainability into practice, several frameworks and tools guide the implementation of environmentally responsible actions in sport event management. The circular economy model challenges the traditional “take–make–waste” approach by promoting continuous resource reuse and waste reduction (23). Its core principles, responsible resource use, material optimization, and impact reduction can be applied to sport through modular venue design, the implementation of reusable materials, and circular procurement strategies. The 6R model builds on this concept by incorporating six actions: refuse, reduce, reuse, rethink, recycle, and recover (24). Taken together, these models support the development of circular systems that enhance efficiency while lowering the overall environmental footprint.

International standards provide a structured foundation for promoting sustainability. The Global Reporting Initiative (GRI) offers guidelines for transparent reporting of environmental and social performance (25), while ISO 20121 defines requirements for sustainable event management systems, ensuring responsible resource use and active stakeholder participation throughout the event lifecycle (26, 27). Moreover, the Life Cycle Assessment framework (ISO 14040:2006) enables the evaluation of environmental impacts across all stages of production and operation, supporting evidence-based decision-making (28).

Integrating these models and standards enables event organizers to design comprehensive strategies that lower ecological footprints, improve operational efficiency, and align with global sustainability objectives. However, their practical application often depends on contextual elements such as event size, technological capacity, and local policy frameworks. Ultimately, the effectiveness of sustainable management relies on shared responsibility, where organizers, participants, and institutions work together to ensure that sport events not only reduce environmental impact but also support broader social and ecological well-being.

#### 2.1.5 Sustainable practices in sports events

Practical examples of these principles can be seen in recent mega-events that have integrated sustainability into their planning and operations. The *FIFA World Cup Qatar 2022* adopted multiple strategies to address its environmental challenges, including the use of renewable energy, water recycling systems, and the construction of modular stadiums designed for disassembly and reuse (39). This approach enabled the tournament to significantly reduce carbon emissions and material waste while setting new standards for sustainable infrastructure in sport. Although, this events remains highly controversial.

Beyond infrastructure, the event also implemented a comprehensive sustainability management system certified under ISO 20121, covering supplier selection criteria, waste management protocols, and carbon monitoring across all 64 matches and eight host stadiums (42). Sustainability requirements were embedded directly into procurement contracts, making environmental compliance a condition of participation rather than an optional commitment. These practices illustrate how international certification frameworks can be operationalized at scale, providing a concrete reference point for the management models examined in this study.

Similarly, the *Paris 2024 Olympic Games* have embedded sustainability at the core of their design and management philosophy. The event aims to cut carbon emissions by 55% compared to previous editions through the use of renewable energy, bio-based construction materials, and extensive recycling initiatives (29). Measures also include biodiversity protection, sustainable food sourcing, and the creation of long-term community infrastructure such as the Athletes’ Village, which will be converted into eco-friendly housing after the Games (30).

The Paris 2024 Games also established a collaborative governance model involving over 400 partners committed to shared sustainability criteria, spanning procurement, transportation, and legacy planning (30). This multi-stakeholder architecture required the coordination of organizers, public authorities, sponsors, and suppliers around a common environmental framework, a model of shared responsibility that directly illustrates the collaborative dimensions examined in Section 3.3 of this study. Taken together, these two cases demonstrate that effective sustainability management in sport events is neither purely technical nor solely organizational, but depends on the simultaneous alignment of tools, governance structures, and cultural commitment.

Based on these two cases, several recommendations emerge for sport event managers. First, sustainability certification such as ISO 20121 should be integrated into procurement processes from the earliest planning stages, rather than applied retroactively. Second, carbon reduction targets should be embedded into the governance structure itself, with shared accountability across all partner organizations. Third, legacy planning, converting temporary infrastructure into long-term community assets, should be considered a core sustainability outcome rather than an optional add-on.

#### 2.1.6 Identified research gaps

Despite the growing academic interest in environmental sustainability within sport, the existing literature remains fragmented and often provides limited practical guidance for event organizers. Previous research has mainly focused on identifying environmental impacts rather than developing integrated management frameworks applicable across different sporting contexts. Furthermore, there is limited empirical evidence on how sustainability tools, such as circular economy principles, Life Cycle Assessment (LCA), or ISO 20121, are implemented in practice.

To address these gaps, this study explores how environmental sustainability can be effectively integrated into sport event management. It examines strategies and management models aimed at reducing ecological impact while maintaining operational efficiency and promoting stakeholder collaboration.

### 2.2 Presentation of the research question

Sports events are complex, resource-intensive events with significant environmental impacts. Although sports events can promote sustainability, organizers often face difficulties in implementing comprehensive environmental strategies due to operational complexity and competing priorities. This results in critical challenges:

Environmental challenges:

- High carbon emissions due to energy consumption, transportation, and construction of temporary venues.
- Large-scale waste generation, including single-use plastics, event decorations, and discarded promotional materials.

Organizational and managerial barriers:

- Limited integration of environmental sustainability principles into event planning.
- Insufficient managerial expertise in sustainability management.
- Lack of access to clear sustainability specifications and frameworks.

Systemic barriers:

- Fragmented stakeholder involvement, complicating sustainability implementation.
- Inconsistent enforcement of international sustainability standards.
- Perceived high costs of implementing sustainability tools and certifications.

*Which solutions can be implemented to reduce the environmental impact of sports events while ensuring operational effectiveness and promoting long-term sustainability in event management?*

### 2.3 Presentation of hypothesis

Hypothesis 1: *The identification of specific environmental challenges linked to sports events, such as emissions, waste production, and resource depletion, facilitates the implementation of tailored sustainability measures, improving environmental performance and reducing ecological harm*.

Hypothesis 2: *The adoption of key sustainability tools such as Life Cycle Assessment (LCA), circular economy models, and environmental certification frameworks (e.g., ISO 20121) enhances the environmental performance of sports events*.

Hypothesis 3: *Collaborative efforts among stakeholders, including event organizers, sponsors, suppliers, local communities, and policymakers, are essential for effectively implementing environmental sustainability specifications in sports events*.

Given the format of this article and the methodology we used (9 interviews), it is nonetheless essential to view our research as “exploratory” rather than “confirmatory.” Indeed, much more ambitious and rigorous protocols would be necessary to lend greater weight to our findings. Nevertheless, we have attempted to identify hypotheses based on the literature review we conducted.

### 2.4 Methodology

#### 2.4.1 Research design

This research adopts a qualitative approach combining primary and secondary data to develop a framework of best practices for sustainability in sport event management. The integration of both methods allows for a comprehensive understanding of current practices, challenges, and solutions related to environmental sustainability. This study adopts an interpretivist approach, viewing sustainability practices as socially constructed and shaped by context. It therefore seeks to understand them through the experiences of practitioners (41). This perspective supports the use of semi-structured interviews as the main data collection method, as they enable an in-depth exploration of individual views and organizational realities that would be difficult to capture through quantitative approaches.

#### 2.4.2 Data collection

##### 2.4.2.1 Primary data

Primary data were collected through semi-structured interviews with sport event organizers and sustainability experts allowing for open discussion while maintaining thematic consistency. A semi-structured interview guide was developed prior to data collection, organized around five thematic areas reflecting the study's objectives: professional background and sustainability role; environmental challenges in event management; current practices and related barriers; stakeholder collaboration; and future perspectives.

This structure allowed for open discussion while maintaining thematic consistency, supporting the identification of real-world challenges, innovative practices, and institutional barriers in sustainability implementation (31).

##### 2.4.2.2 Secondary data

Secondary data were drawn from a wide range of academic and institutional sources, including sustainability frameworks and reports published by major international sports governing bodies and event organizations. These materials provided the theoretical and policy foundations necessary to interpret and validate the primary findings.

The analysis focused on key reference documents such as the *UEFA Circular Economy Guidelines* (2023), the *Commonwealth Games Sustainable Sourcing Code* (2022), the *ECA Sustainability Strategy* (2024), the *FIFA World Cup Qatar 2022 Sustainability Strategy*, and the *IOC Sustainable Sourcing* Guides (2019–2023). Together, these frameworks offered concrete examples of environmental management, procurement policies, and sustainability reporting practices applied at different event scales, thereby reinforcing the analytical depth and external validity of the study (32).

#### 2.4.3 Sampling and participants

Participants were selected through purposive sampling, ensuring the inclusion of individuals with recognized expertise in sustainability and event organization. The final sample comprised nine professionals representing diverse organizations, including international federations, event committees, sport clubs, and consultancies. This diversity enabled the identification of both common patterns and context-specific variations in how sustainability is interpreted and applied. However, the absence of perspectives from fans, sponsors, and suppliers is acknowledged as a limitation, as these actors play an increasingly central role in the ecological footprint and social impact of sport events.

This point regarding demand is crucial here, and we will return to it after analyzing the results, in a section that addresses the limitations of our research and the need to conduct a quantitative study of demand. This approach will follow a more marketing-oriented logic, in which the researcher analyzes any potential disconnect with the strategy deployed by the supply side to “green” its policy, particularly in the context of organizing sporting events.

#### 2.4.4 Data analysis

All interviews were conducted remotely via Zoom, recorded with participants' consent, and transcribed verbatim. They were carried out in English, Italian, and French and subsequently translated into English to ensure uniformity during the analysis phase. A thematic analysis approach was employed to examine the qualitative data, combining inductive and deductive coding strategies to identify key themes. The analysis focused on three main dimensions: the identification of environmental impacts related to energy use, waste, and resource management; the evaluation of tools and strategies implemented to mitigate these effects; and the examination of collaboration mechanisms among stakeholders. Each participant was anonymized and coded (E1–E9) to maintain confidentiality, and the resulting themes were cross-referenced with secondary sources to ensure consistency and validity.

The qualitative data were imported into NVivo to facilitate the coding process and thematic analysis (Nvivo 14 Release 14.24.0), with the emerging themes subsequently reviewed and validated by the authors to ensure interpretive consistency (Table 1).

**Table 1:** Summary of the interviewees.

Interview	Organization	Job position	Date	Duration
E1	Non profit association involved in ecology (Italy)	Co-founder and President	22/01/2025	45 min
E2	Sport federation (England)	Senior Event Manager	20/01/2025	40 min
E3	Company managing arenas (international)	Box Office Assistant	21/01/2025	29 min
E4	Managing clubs association (Europe)	Head of Sustainability	18/12/2024	44 min
E5	International federation	Sustainability Manager	10/01/2025	38 min
E6	Agency specialized in sustainability (International)	Communications Director	08/01/2025	34 min
E7	National federation (Switzerland)	Tournament and sports equipment manager	23/12/2024	35 min
E8	International Federation	Sustainability Director	19/12/2024	48 min
E8	Sport event organizer (Switzerland)	Tournament Manager	06/01/2025	29 min
E9	Sport federation (England)	Senior Event Manager	24/01/2025	43 min
				Average: 40 min

#### 2.4.5 Sample evaluation

The diversity of roles represented, including sustainability managers, event organizers, and consultants, ensures a plurality of opinions and expertise, providing in-depth and specialized perspectives in the field. Additionally, the sample covers globally significant sports such as football, hockey, tennis and skiing, as well as more niche disciplines like golf and cricket. This variety balances the organizational dynamics of large-scale events, such as the Ski World Cup and the FIFA World Cup, which require years of planning and collaboration among numerous teams, with more specific and smaller-scale contexts, such as the ATP 250 Swiss Open Gstaad tennis tournament. The second one, focused mainly on local fans and managed by a much smaller team, demonstrates how smaller events are organized. Additionally, the inclusion of a professional specializing in an organization focused on the conscious restoration of environmental debt outside of sports events adds a valuable dimension to the study. This perspective provides insights into broader sustainability practices and strategies that can be adapted to sports contexts. The inclusion of a participant in an operational venue management role also provides a ground-level perspective on how sustainability decisions are experienced at the point of implementation, complementing the strategic vision offered by senior managers. This variety of sports and organizational scales offers a nuanced and representative view of the realities of sustainability in sports events, providing a comprehensive understanding of the different needs and solutions adopted in various contexts.

However, it is important to highlight some gaps in the sample: perspectives from stakeholders working in fan engagement, athletes or community sectors, as well as those of sponsors directly involved in sustainability, are missing. These roles are crucial for the success of an event, considering that fans constitute the majority of the event's audience and impact, while sponsors and suppliers make the event's execution possible. Their exclusion limits the study, which thus remains only partially analyzed. Incorporating these stakeholders would help address the gaps in the study, offering a more comprehensive and integrated view of sustainability dynamics in sports events.

#### 2.4.6 Integration of primary and secondary data

The integration of both primary and secondary data enabled methodological triangulation, strengthening the overall robustness of the analysis. Primary data provided first-hand perspectives grounded in practical experience, while secondary sources situated these insights within established frameworks such as the circular economy, Life Cycle Assessment (LCA), and ISO 20121 standards. This approach ensured that the study's conclusions were both empirically supported and theoretically informed.

#### 2.4.7 Analytical dimensions

The evaluation of secondary data was structured around two main dimensions of sport event management: logistics and infrastructure, which includes transportation, energy, technology, and materials; and experience and stakeholder management, which covers areas such as catering, merchandising, accommodation, and communication. For each dimension, two analytical lenses were adopted. The first examined sustainable practices, focusing on resource efficiency and procurement, while the second explored end-of-life management, including reuse, recycling, and waste minimization processes.

## 3 Results and managerial implications

The results of this study confirm that advancing environmental sustainability in sports events depends on three interconnected dimensions: the identification of event-specific environmental challenges, the adoption and contextualization of sustainability tools supported by education, and the establishment of collaborative management frameworks. Together, these factors transform sustainability from an aspirational concept into an operational practice grounded in data, adaptability, and shared responsibility. Across the nine interviews, a notable degree of convergence emerged on the two dominant impact categories, transportation and food waste, regardless of organizational scale or sport type. Divergence was more pronounced regarding sustainability tools and their feasibility, with participants from large federations favoring certification frameworks and those from smaller events prioritizing practical, low-cost measures.

### 3.1 Understanding and managing environmental challenges

Making a sporting event more sustainable does not mean achieving zero emissions or generating no waste, such a goal is unrealistic. Every activity leaves an environmental footprint. The realistic objective is not perfection but continuous improvement, adaptation, and education aimed at minimizing impact.

The study highlights that awareness of the need for change is now widespread across the sports sector, particularly within international federations. These organizations are leading the way by developing sustainability strategies, guidelines, and educational tools. Importantly, they promote open dialogue and transparency, fostering collaboration rather than competition. This culture of shared learning, still uncommon in other industries, represents one of the most powerful drivers of change.

However, significant barriers remain, particularly cultural and organizational resistance to change, often expressed as financial limitations. As E8 notes, “*it is very cultural, people are used to working in a certain way with certain suppliers. Breaking that habit is difficult*” (E8, 2024). Overcoming these challenges requires leadership, vision, and cooperation. Sustainability in sport should therefore be viewed not as a burden but as an opportunity to innovate, educate, and build a more equitable future.

The evidence confirms that recognizing environmental impacts is fundamental to reducing them. Two primary sources of impact dominate most events. As E8 notes, transportation alone “*accounts for 80% of the impact of any event*” (E8, 2024), while food and beverage waste represents the second major challenge (E4, 2024; E5, 2025). At smaller scales the pattern holds: as E9 reported, “*transportation is really the biggest contributor to our negative sustainability impact, with about 70% of people arriving by car*” (E9, 2025). These, however, differ in their degree of controllability. Transportation largely depends on local infrastructure and public policy, whereas food procurement, catering, and waste prevention lie directly within the organizer's sphere of influence. In this regard, E1 noted that the priority must be clear: “*first of all, start from the reduction of waste production*” (E1, 2025). This principle of reduction at source, rather than compensation after the fact, underpins the entire approach to impact management discussed in this study.

Identifying where control exists allows for targeted action, for example, choosing local suppliers, reducing packaging, and optimizing catering operations. Meanwhile, transportation issues can be mitigated through partnerships with municipalities or sponsors that promote public transport and carpooling initiatives.

Importantly, impacts vary across contexts. A ski event in the Alps faces different challenges than a football tournament in an urban stadium. Each event takes place within a unique cultural, geographical, and infrastructural setting, requiring tailored strategies rather than uniform solutions. As E5 emphasizes, “*it's too difficult to put a regulation in place, a regulation for a country as Finland can never be applied the same way in a country as Slovenia*” (E5, 2025).

This contextual flexibility, understanding local challenges, constraints, and opportunities, is what makes environmental assessment essential to effective sustainability management.

### 3.2 The role of sustainability tools and the energy budget framework

Sustainability tools provide the structural foundation for planning, implementing, and evaluating environmental performance in sports event management. Certifications such as ISO 20121 and ISO 14001 guide organizers toward systematic management practices that align with internationally recognized standards, promoting transparency, accountability, and continuous improvement. Similarly, circular economy models encourage material reuse, recycling, and waste reduction, while carbon calculators enable the quantification of emissions and the identification of priority areas for mitigation. Examples such as the FIS Carbon Calculator or UEFA's Circular Economy Guidelines demonstrate how structured methodologies can support measurable and verifiable sustainability outcomes. The practical weight of these tools becomes evident when sustainability criteria are embedded in procurement decisions: as E4 observed, “*if you don't have this certification or you can't approve that you have these practices, we don't consider you, or we give you a lower score*” (E4, 2024).

The effectiveness of these tools, however, depends on their contextual adaptation and the users' understanding of their purpose. What may be effective for a large international championship is not necessarily suitable for a smaller local tournament. Tools must therefore be flexible and scalable, allowing their integration across events of different sizes and resources. Smaller organizations often encounter challenges in adopting such systems due to financial limitations, insufficient technical expertise, or lack of access to training. In these cases, simplified frameworks or collaborative support from governing bodies can enable more inclusive implementation. The stakes of non-adoption are significant: as E2 warned, organizations that fail to keep pace with sustainability standards “will be left behind” (E2, 2025). Adopting sustainability tools is therefore not a question of size or resources alone, but of institutional will.

Despite their value, tools alone cannot drive meaningful change. Real progress depends on education, awareness, and creativity. Training event staff, volunteers, and participants fosters a culture in which sustainability becomes an embedded organizational value rather than a procedural obligation. As E4 illustrated, this requires a structured approach: at the FIFA World Cup, organizers set up “*a huge training system identifying which categories of subjects needed to be trained on what, from volunteers to catering or food and beverage staff*” (E4, 2024). This principle extends beyond mega-events: as E7 stated, “*educating athletes is key*” (E7, 2024). Education, however, is not only about content but also about tone and framing. As E6 suggested, sustainability communication should be “less front-page and more focused on behavioral change” (E6, 2025). When education supports technical instruments, sustainability evolves from a compliance requirement into a shared ethos guiding decision-making at every stage of event management.

Environmental compensation measures represent another crucial dimension of sustainability tools, although their role must be assessed critically. Complete environmental neutrality remains unattainable, as every event inevitably generates emissions, waste, and ecological disruption. As a result, organizers frequently introduce compensation initiatives such as carbon offsetting or reforestation programs. However, offsetting emissions in remote locations does not repair the environmental degradation occurring at the event site. Effective compensation strategies should therefore prioritize local ecological restoration and regeneration. E8 highlights the importance of working with forestry services to mitigate environmental impacts: “*If trees need to be cut down for safety reasons in a race area, it's essential to find local activities to compensate for this impact, ideally double what was damaged*” (E8, 2024). This place-based approach to compensation underscores that ecological repair must be rooted in the same context where the damage occurs. Moreover, while social initiatives, such as education or community engagement, offer important societal benefits, they cannot substitute for direct environmental repair. Sustainable event management must combine emission reduction at the source with scientifically validated, localized compensation practices that restore ecological balance.

Leadership and communication play a decisive role in enabling these processes. Federations and governing bodies can accelerate sustainable transformation by promoting knowledge sharing, capacity-building, and continuous feedback between events. E4 describes this multi-layered collaboration in the context of the FIFA World Cup, where several entities work together to ensure the event's success: “*you always have FIFA, a local organizing committee, and the supreme committee, which is instead a public company*” (E4, 2024). This governance structure illustrates how sustainability responsibilities must be clearly distributed across institutional levels to be effective. The establishment of vertical and horizontal communication systems ensures that lessons learned at one level inform practices at another, strengthening the overall coherence and efficiency of sustainability efforts.

Practical sustainable choices can include prioritizing reusable materials, locally sourced products, and items with longer lifecycles. Among the most innovative approaches identified in this study is the concept of the energy budget, which introduces a new paradigm for resource management in sports events. The energy budget treats energy and environmental resources as finite and valuable assets, comparable to financial capital, requiring systematic planning, monitoring, and optimization. Just as a financial budget allocates funds to specific categories, an energy budget distributes “energy credits” across operational areas such as transport, catering, and venue operations. The concept mirrors personal financial planning: as E1 proposed, events should adhere to an energy budget in the same way individuals manage daily expenses, “*you have your environmental energy budget, you scan the product, and the app tells you your budget is Y. The pasta costs Y, you take it or don't take it*” (E1, 2025). This approach enables organizers to visualize trade-offs, identify inefficiencies, and prioritize resource allocation according to sustainability objectives without compromising event quality.

By quantifying and managing energy consumption, the energy budget framework encourages mindful decision-making and fosters accountability throughout the event lifecycle. It transforms sustainability from a conceptual ambition into a measurable management practice grounded in data and performance indicators.

Ultimately, progress toward sustainability in sports events depends not on achieving perfect solutions but on making informed, responsible choices. Each decision, whether related to transport, procurement, or resource use, contributes incrementally to minimizing environmental impact. The integration of robust tools, education, and innovative management frameworks such as the energy budget provides a realistic and effective pathway toward a more sustainable future for sports event management.

### 3.3 Collaborative management and shared responsibility

Collaboration represents the cornerstone of sustainable event management. Environmental responsibility in sport depends on the coordinated efforts of multiple actors, including organizers, sponsors, suppliers, local authorities, policymakers, and spectators. Each stakeholder plays a specific role: organizers define strategic objectives, sponsors provide financial and communication support, suppliers ensure the use of sustainable materials and production methods, policymakers establish regulatory frameworks, and fans influence outcomes through their behaviors and participation choices.

Evidence shows that collaborative initiatives are already generating tangible results. Joint actions between organizers and suppliers have advanced sustainable procurement, while partnerships with local authorities have improved waste management and carbon monitoring. Engagement campaigns targeting fans have also proved effective in promoting responsible behaviors, such as waste sorting and reduced resource use. Such diffusion of good practices can happen organically: as E5 observed, once one organizer introduced branded bicycles for team transportation, “*now every organizer tried to put bikes in front of the team hotels so the teams can use it: because they saw the teams are using it*” (E5, 2025). These examples demonstrate that when cooperation is structured and goal-oriented, sustainability outcomes become more significant and enduring.

Nevertheless, collaboration also presents notable challenges. Conflicting priorities, communication barriers, and limited stakeholder engagement can hinder coordination and slow progress. Overcoming these limitations requires building mutual trust, ensuring transparency, and establishing shared accountability among all participants. As E2 noted, change is already underway: “*we are starting to put forward, within our new contracts, elements of sustainability so that they need to meet certain criteria*” (E2, 2025). Progress, in this sense, depends as much on cultural alignment as on structural frameworks. Federations and governing bodies play a crucial role in this process by developing frameworks that foster cooperation, knowledge exchange, and consistent sustainability reporting across events.

Collaboration should not be understood merely as logistical coordination but as a cultural commitment embedded in the identity of sport. Sustainability becomes most effective when it is treated not as a source of competitive advantage but as a shared responsibility and collective mission. As E6 noted, effective collaboration requires demonstrating mutual benefit: “*you need to show how it benefits whoever they’re working with, not just because of the sustainable aspect but also financially or operationally*” (E6, 2025). By fostering open communication, creating learning platforms, and promoting cross-event exchanges of best practices, the sports sector can turn environmental challenges into opportunities for innovation, leaving a legacy that extends well beyond individual events.

### 3.4 Managerial implications

The findings of this study provide concrete managerial insights for integrating environmental sustainability into sport event organization. Three main areas of action emerge: the design of context-specific ecological strategies, the preventive evaluation of environmental challenges before event implementation, and managerial support in achieving environmental certifications.

First, the results highlight the importance of developing ecological strategies tailored to the specific characteristics of each event. A one-size-fits-all approach is ineffective, as environmental priorities differ according to context, scale, and available infrastructure. Event organizers should therefore design flexible sustainability plans that address the unique challenges of each setting, whether related to transportation emissions, energy consumption, or waste generation. Applying frameworks such as the circular economy, the energy budget model, and ISO 20121 standards enables managers to balance ecological goals with operational feasibility, ensuring that sustainability becomes a structural element of event management rather than an additional constraint.

Second, the study underlines the need for preventive assessment of environmental risks prior to event organization. Conducting early-stage evaluations allows managers to anticipate and mitigate impacts before they occur, facilitating more efficient resource allocation and reducing the need for corrective measures during the event. As E3 underlined, “*you need to know where you can implement things and where they are going to work*” (E3, 2025). This proactive approach supports long-term sustainability by embedding environmental considerations at the outset of strategic and logistical planning. Preventive assessment also enhances stakeholder coordination, ensuring that sustainability responsibilities are clearly distributed among organizers, suppliers, and local authorities.

Third, the research emphasizes the importance of managerial support in pursuing environmental certifications and labels. Obtaining certifications such as ISO 20121 or national environmental labels not only formalizes sustainability commitments but also reinforces accountability, transparency, and external recognition. As E8 noted, even simple operational decisions can generate significant gains: “*switching to green electricity often just means changing a contract in 90% of cases*” (E8, 2024). Managers involved in certification processes are better equipped to implement structured monitoring systems, standardize performance indicators, and align operations with international benchmarks. Institutional support from federations and governing bodies can further assist organizers in navigating certification procedures and accessing specialized training opportunities.

Taken together, these implications outline a practical roadmap for strengthening sustainability performance in sport event management. By developing context-sensitive strategies, implementing preventive assessments, and institutionalizing environmental certification, sport organizations can operationalize sustainability in a measurable and lasting way. These managerial implications encourage a shift from isolated green initiatives toward a systemic, data-driven model of sustainable event management capable of generating both ecological and organizational value. Underpinning all of this is a culture of continuous reflection: as E9 recalled, a seminar speaker simply asked “what are you doing? What aren't you doing?” (E9, 2025), a question that captures the self-critical mindset essential to lasting progress. Sustainability, in this sense, is not a destination but a practice of ongoing questioning and adaptation.

## 4 Discussion: limitations and future research

Given the exploratory and qualitative nature of this study, the three hypotheses are not subjected to statistical confirmation but are assessed through an abductive reasoning approach (33, 44), drawing on the convergence of interview data and secondary sources.

Hypothesis 1 is strongly supported. The results confirm that identifying event-specific environmental challenges is a prerequisite for effective sustainability action. Transportation and food waste consistently emerge as the dominant impact categories across all contexts, yet the data reveal that what distinguishes effective management is not the recognition of these categories *per se* but the ability to differentiate between controllable and non-controllable impacts (E9, 2025; E5, 2025). This contextual diagnosis enables targeted interventions and prevents the misallocation of resources toward areas outside the organizer's sphere of influence.

Hypothesis 2 is partially supported. Sustainability tools, including ISO 20121 certifications, circular economy frameworks, and the energy budget model introduced in this study, provide structured methodologies for planning and reducing environmental impact (E4, 2024; E1, 2025). However, the data consistently show that tool effectiveness is contingent on contextual adaptation, user education, and organizational will. Smaller organizations in particular face significant barriers to adoption, suggesting that the tools' theoretical potential is not automatically translated into practice without institutional support (E2, 2025).

Hypothesis 3 is strongly supported. No single actor can address the environmental challenges of sports events in isolation. The findings demonstrate that collaboration, when grounded in demonstrated mutual benefit rather than abstract sustainability goals, generates self-reinforcing dynamics that accelerate progress across the sector (E6, 2025; E5, 2025). The contractual mechanisms and peer-to-peer diffusion of best practices observed in the data suggest that collaborative frameworks are among the most operationally effective drivers of change available to event organizers.

While these findings offer meaningful empirical support for all three hypotheses, they also underscore the exploratory nature of this research. More rigorous confirmation would require larger, mixed-method studies. The following section addresses the study's limitations and outlines directions for future inquiry.

This study offers valuable insights into environmental sustainability within sports event management but also presents several limitations that define avenues for future research. Sport ecology remains an emerging field, and while the literature on sustainability in sport has expanded, the operationalization of ecological principles into practical models is still underdeveloped. The analysis focused primarily on the environmental dimension of sustainability, while the social and economic pillars, equally essential for a holistic understanding, were only partially addressed. Integrating these dimensions would provide a more comprehensive view of how ecological goals can coexist with social inclusion and economic feasibility.

From a methodological perspective, the qualitative approach based on semi-structured interviews provided depth but limited generalizability. Future research could broaden the empirical base through larger, mixed-method studies combining qualitative insights with quantitative surveys across sports, countries, and event scales. Additionally, direct observation of live events could capture the dynamic and situational challenges of implementing sustainability strategies, an aspect not fully explored in this work.

Future research should address three key directions: the economic dimension of sustainability, assessing both the costs and the long-term financial benefits of green practices; the behavioral dimension, examining how communication and fan engagement can promote more sustainable consumption and mobility choices; and the balance between ecological and social impacts, evaluating whether the positive societal outcomes of sport can offset its environmental footprint.

By addressing these areas, future research can contribute to the development of an integrated, evidence-based framework that connects environmental responsibility with social value and economic viability, thereby advancing the transition of sport event management toward genuinely sustainable practice.

The aim of our article was to bridge the gap between grey literature and consultancy reports.

While remaining humble, we believe we have partially fulfilled our initial ambitions, but it seems logical and useful to expand this research, which can be considered exploratory.

If we wish to further the research topic we have attempted to explore, it would be useful to develop an ambitious research program in the future.

From a methodological standpoint, our article has a number of limitations that could be addressed by adopting a more rigorous approach and design.

First, a thorough analysis must be conducted after rigorously formulating the hypotheses.

A more conclusive demonstration can only be achieved by using a larger sample of federations/organizations/cities/sport events organizers that is more representative of the highly heterogeneous nature of these institutions.

Within each of those institutions, the case study methodology (34) could be used, which would involve interviewing at least four different stakeholders holding key managerial positions within the organization. Within each organization, the process should then be replicated with individuals holding the same position. Ideally, four to six cases should be studied. If these interviews are supplemented with a few additional interviews with consultants/experts in the field and in technology, this would result in a minimum of 16–24 interviews.

If we could conduct several rigorous case studies—with tangible points of comparison and in accordance with this case study protocol—we would then have much richer data with which to evaluate the environmental policies implemented by organizations.

Also, the use of Yin (35), Dubois and Gadde (33) strengthens our methodology by providing a balance between structural rigor and theoretical flexibility:

Yin (35) is the gold standard for a formal, systematic approach to case studies; our research design would benefit from a better validity and reliability through strict protocols and data triangulation. In contrast, Dubois and Gadde (33) introduced “systematic combining,” an abductive approach that allows for a dynamic interplay between theory and empirical observation. By combining them, we could have a methodology which is both scientifically disciplined and analytically adaptive, enabling us to refine existing theories while maintaining a robust, defensible framework. This dual foundation is particularly effective for making our study a more credible piece of evidence and a source of new conceptual insights.

But the ultimate arbiter will undoubtedly be the consumer/spectator/viewer, whom we will need to survey.

Therefore, a new topic should be considered, which is consumer behavior.

As mentioned earlier, our study measures organisational intentions and practices on environmental issues. But, a risk exists, which is “greenwashing”. Greenwashing is the deceptive marketing tactic of portraying a company, product, or service as more environmentally friendly or sustainable than it actually is. It involves making misleading, exaggerated, or false claims to capitalize on consumer demand for green products, often hiding damaging environmental impacts. This strategy is often combined with a “social washing” strategy.

Many researchers have already studied this in sport (36–38), and testing this concept would give credibility to further research.

Therefore, if we are to address this issue properly, it is obviously necessary to draw on the extensive literature in marketing and consumer behavior, paying particular attention to greenwashing perceptions.

In fact, all sports organizations conduct favorable environmental impact studies aimed at demonstrating their responsibility, their respect for nature in all its forms, and their consideration of the most favorable impact indicators possible. Since the 1990s, the IOC and FIFA, for example, have been conducting such studies on their flagship events to showcase “progress made,” while “humbly” emphasizing the need to strive to “do better” in the future.

Ultimately, to properly address the research question regarding consumers, their perceptions, and the effectiveness of the environmental policy/strategy used, it seems essential to conduct a quantitative study among fans, spectators, and television/social media viewers. Here are the key elements that will enable us to conduct more relevant and comprehensive research, so that the exploratory findings we have identified can be confirmed or refuted.

## 5 Conclusion

Sport is a key driver for building healthier, happier, and more connected societies. It embodies values of resilience, determination, and respect, inspiring passions, dreams, and goals. Sporting events celebrate these values, uniting fans across arenas and circuits, while showcasing the sacrifices and triumphs of countless athletes. However, while these events bring joy and inspiration, they also significantly impact the environment.

By their very nature, sports events are temporary, designed to occur once and then dissolve. This characteristic mirrors the concept of “single use,” which is unsustainable in the long term. A cultural shift is urgently needed, one that prioritizes environmental responsibility in event planning and execution. This shift begins with education, the use of appropriate tools, and the involvement of the right people.

The most pressing challenges identified are transportation and waste. As sport relies entirely on the environment, snow for skiing, water for aquatic sports, clean air for outdoor events, it must adapt to protect these essential resources. Achieving zero environmental impact may be unrealistic, but significant reductions are achievable through strategic planning and innovation.

Sports events, beyond being platforms for realizing dreams and inspiring future generations, must serve as examples of sustainable practices for industries and societies. They have the potential to demonstrate that change is not only necessary but entirely possible. By adopting sustainable management practices, integrating circular economy models, and fostering collaboration among stakeholders, sports events can transition from being sources of environmental harm to examples of positive change.

This research underscores the importance of leadership, education, and collaboration in achieving sustainability in sports events. It highlights that while challenges remain, the opportunity to transform these events into drivers of environmental and social change is immense. Sporting events, as symbols of aspiration and achievement, can lead the way toward a more sustainable future, proving that excellence on the field can coexist with environmental responsibility beyond it.

## Data Availability

The original contributions presented in the study are included in the article/Supplementary Material, further inquiries can be directed to the corresponding author.
